# Combining 'omics and microscopy to visualize interactions between the Asian citrus psyllid vector and the Huanglongbing pathogen *Candidatus* Liberibacter asiaticus in the insect gut

**DOI:** 10.1371/journal.pone.0179531

**Published:** 2017-06-20

**Authors:** Angela Kruse, Somayeh Fattah-Hosseini, Surya Saha, Richard Johnson, EricaRose Warwick, Kasie Sturgeon, Lukas Mueller, Michael J. MacCoss, Robert G. Shatters, Michelle Cilia Heck

**Affiliations:** 1 Plant Pathology and Plant-Microbe Biology Section, School of Integrative Plant Sciences, Cornell University, Ithaca, New York, United States of America; 2 Boyce Thompson Institute, Ithaca, New York, United States of America; 3 Department of Genome Sciences, University of Washington, Seattle, Washington, United States of America; 4 Subtropical Insects and Horticulture Research Unit, U.S. Horticultural Research Laboratory, USDA ARS, Fort Pierce, Florida, United States of America; 5 Plant Pathology, University of Florida Citrus Research and Education Center, Lake Alfred, Florida, United States of America; 6 Emerging Pests and Pathogens Research Unit, Robert W. Holley Center, USDA ARS, Ithaca, New York, United States of America; Washington State University, UNITED STATES

## Abstract

Huanglongbing, or citrus greening disease, is an economically devastating bacterial disease of citrus. It is associated with infection by the gram-negative bacterium *Candidatus* Liberibacter asiaticus (CLas). CLas is transmitted by *Diaphorina citri*, the Asian citrus psyllid (ACP). For insect transmission to occur, CLas must be ingested during feeding on infected phloem sap and cross the gut barrier to gain entry into the insect vector. To investigate the effects of CLas exposure at the gut-pathogen interface, we performed RNAseq and mass spectrometry-based proteomics to analyze the transcriptome and proteome, respectively, of ACP gut tissue. CLas exposure resulted in changes in pathways involving the TCA cycle, iron metabolism, insecticide resistance and the insect’s immune system. We identified 83 long non-coding RNAs that are responsive to CLas, two of which appear to be specific to the ACP. Proteomics analysis also enabled us to determine that *Wolbachia*, a symbiont of the ACP, undergoes proteome regulation when CLas is present. Fluorescent *in situ* hybridization (FISH) confirmed that *Wolbachia* and CLas inhabit the same ACP gut cells, but do not co-localize within those cells. *Wolbachia* cells are prevalent throughout the gut epithelial cell cytoplasm, and Wolbachia titer is more variable in the guts of CLas exposed insects. CLas is detected on the luminal membrane, in puncta within the gut epithelial cell cytoplasm, along actin filaments in the gut visceral muscles, and rarely, in association with gut cell nuclei. Our study provides a snapshot of how the psyllid gut copes with CLas exposure and provides information on pathways and proteins for targeted disruption of CLas-vector interactions at the gut interface.

## Introduction

Huanglongbing (HLB) is the most serious disease of citrus. First described in China over 200 years ago, it is now found in many citrus-growing regions around the world, including the United States. Since the initial detection of the disease in in Florida in 2004, the Florida citrus industry has lost over $9 billion in revenue and over 8,000 jobs [1]. HLB infection is associated with infection by the Gram-negative bacterium, *Candidatus* Liberibacter asiaticus (CLas). CLas is transmitted from tree-to-tree by *Diaphorina citri*, the Asian citrus psyllid (ACP), in a circulative manner. In contrast to other circulative plant pathogens which require a systemically infected plant as a source for acquisition, CLas can be horizontally transmitted to psyllid nymphs through the newest plant growth, called flush tissue, in just a single insect generation even in the absence of a systemic tree infection [2]. Horizontal transmission of CLas to psyllid nymphs via the flush facilitates a rapid spread that is unprecedented for insect vector-borne plant pathogens [2]. Especially problematic for growers, the ACP vector spreads CLas throughout the grove during a latency period where the trees remain asymptomatic and the pathogen cannot be detected by molecular methods [2].

Long-term management of HLB using insecticides to minimize psyllid populations is both economically and environmentally unsustainable for the citrus industry, and insecticides have not been sufficient to stop the spread of HLB. There are many factors contributing to the failure of insecticides to manage HLB, including the unique way in which CLas acquisition is linked to the reproductive cycle of the psyllid vector, the migratory ability of the vector, the ability of the vector to survive on alternate host plants (at least transiently), and the inability to apply insecticides on all citrus groves simultaneously. HLB research is made more difficult because CLas is an obligate biotroph and cannot currently be grown in culture. The development of HLB-resistant citrus varieties has been hampered due to lack of strong CLas resistance in commercial citrus varieties and the lengthy time needed to conduct complicated breeding or transgenic approaches [3]. With these factors in mind, methods that disrupt interaction between the ACP and CLas are attractive alternatives.

The ACP is a member of the insect order Hemiptera, which includes other important agricultural pests such as aphids and whiteflies (reviewed in [4]). The ACP feeds on plant phloem sap using its piercing-sucking, stylet mouthparts [5]. A draft genome sequence is available for the ACP in NCBI, although the quality is poor (contig N50 of 34.4kp and scaffold N50 of 109.8kb). Extensive efforts are being made to improve the quality of the genome, as well as its annotation using long-read sequencing and community-driven manual curation (https://citrusgreening.org/annotation/index)[6]. The ACP has three known bacterial endosymbionts: *Candidatus* Profftella armatura, *Candidatus* Carsonella ruddii, and *Wolbachia* [7, 8] as well as other internal, extracellular bacteria [9]. Bacterial symbionts of sap-sucking insects often help the insect compensate for the nutritionally poor diet of phloem sap by providing amino acids that cannot otherwise be synthesized by the insect [10]. While the titer of each endosymbiont correlates positively with one another, they vary in localization within the host and predicted endosymbiotic function [11]. Both Profftella and Carsonella are sequestered in the bacteriome, while *Wolbachia* can be found in the bacteriome as well as somatic and reproductive tissues [12, 13]. Metagenomics predicted that 15% of Profftella’s genes are devoted to the synthesis of a novel polyketide toxin [7]. Profftella is thus predicted to have a defensive role in the ACP [7]. Proteomic analysis found that Profftella proteins related to polyketide synthesis are upregulated when CLas is present, and the production of the polyketide is also altered in the presence of CLas [14]. Carsonella may play a more canonical role of supplementing the insect’s amino acid synthesis. The genome of the Carsonella strain associated with the ACP was recently published, and its gene repertoire indicates that it serves as a nutritional symbiont and possesses genes to synthesize essential amino acids [7], which is consistent with its predicted function in related psyllid species [15]. *Wolbachia* is the most common bacterial symbiont of insects. Its interactions with an insect host can range from parasitic to mutualistic; infection in woodlice (*Armadillidium vulgare*) is parasitic and leads to feminization of males, but provides survival and fecundity benefits in *Drosophila melanogaster* [16, 17], where it has also been implicated in the alteration of iron availability by interfering with expression of iron-binding ferritins [18, 19]. In the ACP, titers of *Wolbachia* vary among populations and correlate with CLas titer within the ACP [11, 20, 21].

Although there are many unknowns regarding how CLas is acquired and transmitted by the ACP vector, the transmission is currently best described as following the circulative pathway that has been described for plant viruses transmitted by aphids and whiteflies [4]. Unlike the majority of these viruses, evidence supports the idea that CLas replicates or accumulates within ACP tissues [22]. Compared to viruses, less is known about how hemipterans acquire and transmit bacterial plant pathogens, but many parallels can be drawn when considering the interactions between hemipterans and the circulative viral pathogens. Like circulative plant viruses, CLas must cross several insect tissues including the gut, the insect’s blood (hemolymph) and the salivary glands prior to transmission to a new tree. Transmission of CLas by adults is more efficient when the bacteria are acquired by nymphs, and proteomic data support the idea that the CLas takes advantage of a suppressed nymphal immune system that was selected to cope with establishment of the beneficial symbionts [23]. The CLas bacterium does not induce appreciable mortality in nymphs and only has a somewhat minor impact on adult longevity [24].

The polarized epithelial cells in the gut represent the first barrier to transmission by the psyllid. Midguts dissected from adult ACP exposed to CLas-infected sweet orange show melanization and molecular signatures of programmed cell death (PCD), including nuclear degeneration and annexin V localization to the cell periphery [25]. This study suggests a model where CLas acquisition into the hemocoel (body cavity) relies on the PCD in the midgut tissue, which is puzzling in light of the fact that CLas has a minor impact on the life history traits of adult insects [24, 26]. The observed PCD in the gut also contrasts with whole body proteome analysis from our lab that shows CLas enhances ACP metabolism [14], and these data align closely with the published behavioral and organismal studies showing neutral and/or positive effects of CLas on the vector. Taken together, these studies indicate that the ACP gut experiences a distinct response to CLas that is not found in other tissues, and this gut response may be critical to our understanding as to why insects do not transmit the pathogen when it is acquired as an adult. In this study, we explored the interactions between CLas and the ACP gut by performing a dual transcriptomic and proteomic analysis of ACP gut tissue dissected from CLas-exposed and unexposed insects. Complemented by confocal microscopy to visualize CLas and *Wolbachia* in the gut, the transcriptome and proteome analysis each gave unique and complementary insights into this critically important vector-pathogen interface.

## Materials and methods

### Insect colonies and gut dissections

Samples were collected from insects reared on either healthy or CLas-infected plants. Four biological replicates from CLas- insects were used for protein extraction, and three biological replicates were used from CLas+ insects.

One to two week old, adult *Diaphorina citri* Kuwayama (Hemiptera: Psyllidae) were obtained from age synchronized colonies maintained on *Citrus medica* (Citron). Psyllids originally came from a colony that was established in 1999 from a field population collected from the United States Horticultural Research Laboratory experimental farm at Fort Pierce, FL [27]. Two separate *C*. *medica* colonies were established from the original colony: One on healthy *C*. *medica* and one on CLas infected *C*. *medica*. Fifty psyllids from each were immediately frozen and used for evaluating the percent that contained detectable CLas based on PCR detection of the CLas 16S rRNA gene [28]. The digestive tracts were resected, immediately placed in RLT buffer from the RNeasy Plus Mini kits (Qiagen, Gaithersburg, MD, USA) and stored frozen until used for RNA and protein isolation. In this paper, samples prepared from insects reared on healthy trees are labeled as CLas- and samples prepared from insects reared on CLas-infected trees are labeled as CLas+, although not all insects in the sample are CLas positive (see Results section).

### RNA isolation

A total of 2000 psyllid guts were divided into replicate samples for RNA isolation. Each sample was a pooled biological replicate comprised of 250 psyllid guts. Total RNA was extracted from four CLas positive and four CLas negative samples of psyllid guts using a Trizol (Invitrogen) extraction.

### RNA library preparation and sequencing

Sequencing libraries were constructed with the Illumina Truseq stranded RNA library preparation kit for each sample. Each library was indexed with a unique adapter and the multiplexed sample was sequenced using Illumina Hiseq 2500 in Rapid Run mode. The 2x150bp paired-end reads were analyzed using FastQC(http://www.bioinformatics.babraham.ac.uk/projects/fastqc/) and screened for sequencing adapters and quality with Trimmomatic [29], version 0.36, java -jar trimmomatic-0.36.jar LEADING:5 TRAILING:5 SLIDINGWINDOW:4:10 MINLEN:60 ILLUMINACLIP:TruSeq3-PE-2.fa:2:30:10).

### Differential expression analysis of RNA sequencing data

The filtered reads were aligned with RSEM [30] and bowtie2([31] rsem-calculate-expression—paired-end—strand-specific—bowtie2—estimate-rspd) to the cDNA transcripts (ftp://ftp.citrusgreening.org/genomes/Diaphorina_citri/annotation/NCBI_GNOMON/NCBI%20Diaphorina%20citri%20Annotation%20Release%20100/i5k/rna.fa.gz) generated by the NCBI Eukaryotic Annotation Pipeline (https://www.ncbi.nlm.nih.gov/genome/annotation_euk/process/, https://www.ncbi.nlm.nih.gov/genome/annotation_euk/Diaphorina_citri/100/) run on the NCBI diaci1.1 genome (ftp://ftp.citrusgreening.org/genomes/Diaphorina_citri/genome/NCBI-DIACI_v1.1/121845_ref_Diaci_psyllid_genome_assembly_version_1.1_chrUn.fa.gz) [6, 32]. All transcripts that had less than one count per million in less than three replicates were also excluded from downstream analysis [33]. The read counts for the filtered transcripts were analyzed using edgeR [34] and DESeq2 [35] to identify differentially expressed genes at FDR < 0.01. The scripts used for this analysis are available at https://github.com/suryasaha/ACP_gut_omics_PlosOne_2017. Differential transcriptomic analysis was performed using DESeq2 [35] and edgeR [34]. A negative binomial Wald test was used with FDR threshold of 0.01 and a Benjamini-Hochberg adjusted p-value threshold of 0.05 in case of DESeq2. We used the exact test for differential expression for negative binomially distributed counts [36] implemented in edgeR with the same FDR threshold used with DESeq2. A 2-fold change (FC) cut off was applied.

### Proteomics sample preparation

Gut tissue from adult psyllids was divided into replicate samples of 250 each and collected using centrifugation and re-suspended in 10% TCA in acetone for a protein extraction method our lab has optimized for insect tissues [37] with the following modifications. Tissue was disrupted for two rounds of 10 sec each using a Branson 250 digital sonifier at 10% total amplitude. Total protein was precipitated at -20°C overnight. The precipitated proteins were reconstituted with proteaseMAX surfactant (Promega, Madison, WI). Protein concentration was quantified using a Bradford Assay and confirmed using densitometry on 10% polyacrylamide gels by comparing to a BSA loading control. Proteins were reduced with 5mM tris(2-carboxyethyl)phosphine at 55°C for 20 min, then alkylated with 33mM methyl methanethiosulfonate at room temperature for 20 min. Each sample containing 200μg of protein was digested using 4μg of trypsin at 37°C overnight. The protein digests were dried using a vacuum concentrator prior to mass spectrometry analysis.

### Analysis of peptide samples by mass spectrometry

Peptide samples were resuspended in 0.1% trifluoracetic acid and 2% acetonitrile. Mass spectrometry analysis was performed using an LTQ-Orbitrap Fusion (Thermo Fischer Scientific) as described in [14].

### Mass spectrometry data analysis

Spectral data were searched against a combined database described previously [14] containing predicted proteins from *D*. *citri*, its endosymbionts Carsonella, Profftella, *Wolbachia* as well as CLas using Mascot Daemon 2.3.2 (Matrix Science, Boston, MA). The database had a total of 33,112 combined insect and microbial sequences as well as 112 common contaminant sequences. The search parameters allowed for fixed methylthio modification and variable modifications (methionine oxidation; asparagine, glutamine deamidation) with a peptide mass tolerance of ±20ppm and fragment mass tolerance of ±0.6Da. A maximum of one missed cleavage was allowed. Raw files were converted to Mascot Generic Format files using MSConvert in Proteowizard and used as input files for Mascot Daemon. Peptides were identified at a 95% threshold with a 0.03% decoy false discovery rate (FDR). Proteins were identified with a 99% threshold and 0.6% decoy FDR with a minimum of 2 matching peptides. A Fisher’s Exact Test was employed to identify proteins significantly differentially expressed in CLas+ compared to CLas- samples using spectral counting. A P-value cutoff of 0.05 and 2-FC were applied.

### Detection and phylogenetic status of *Wolbachia* in healthy and exposed ACP to CLas

To confirm the presence of *Wolbachia*, DNA extracted from single guts was subjected to PCR using 16S genus-specific primers [38] to detect the presence of *Wolbachia*. PCR products were gel-purified from a 1% agarose gel using the QIAquick Gel Extraction Kit (Qiagen). PCR-amplified fragments were sequenced and compared with hemipteran *Wolbachia* sequences deposited in GenBank, using Blastn (http://www.ncbi.nlm.nih.gov/blast).

### Quantitative real time PCR assays for *Wolbachia*

DNA isolated from single ACP guts was subjected to qPCR amplification using SYBR Green reagents and the specific primer sets developed for *ftsZ* gene primers (ftsZ-81) for *Wolbachia*: *ftsZ*-F (5′-AGCAGCCAGAGAAGCAAGAG-3′ and *ftsZ*-R (5′-TACGTCGCACACCTTCAAAA-3’) [39]. Absolute quantification of *Wolbachia* copy number was carried out using a standard curve containing three concentrations of a synthetic plasmid containing the sequence of *Wolbachia* ftsZ as described in [39]. An F-test comparing the variance in *Wolbachia* copy number between CLas + and CLas—samples was conducted using Microsoft Excel.

### Fluorescent imaging

Adults ACP were reared on CLas-infected or healthy sweet orange (*Citrus sinensis*) prior to fluorescent *in situ* hybridization (FISH) assays. FISH was performed as previously described [40]. Briefly, specimens were fixed in Carnoy's fixative (chloroform-ethanol-glacial acetic acid, 6:3:1, vol/vol) for 5 min following gut dissection in 1x PBS (phosphate-buffered saline) and hybridized overnight in hybridization buffer (20 mM Tris-HCl, pH 8.0, 0.9 M NaCl, 0.01% [wt/vol] sodium dodecyl sulfate, 30% [vol/vol] formamide) containing 10 pmol fluorescent probes per ml. For specific targeting of 16S *Wolbachia* and CLas, DNA probes (5′-cy5CTTCTGTGAGTACCGTCATTATC-3′) (29) and (5′- cy3CATTATCTTCTCCGGCG -3′) were used respectively. Nuclei were stained with 4′,6′-diamidino-2-phenylindole (DAPI; 0.1 mg ml−1). The stained guts were mounted in hybridization buffer and viewed under a Leica TCS-SP5 (Leica Microsystems Exton, PA USA) confocal microscope. At least 59 CLas+ guts were viewed under the microscope to confirm reproducibility. Specificity of detection was confirmed using no-probe and CLas- controls.

## Results

### The transcriptome and proteome are coordinately regulated in response to CLas

**Fig 1:**
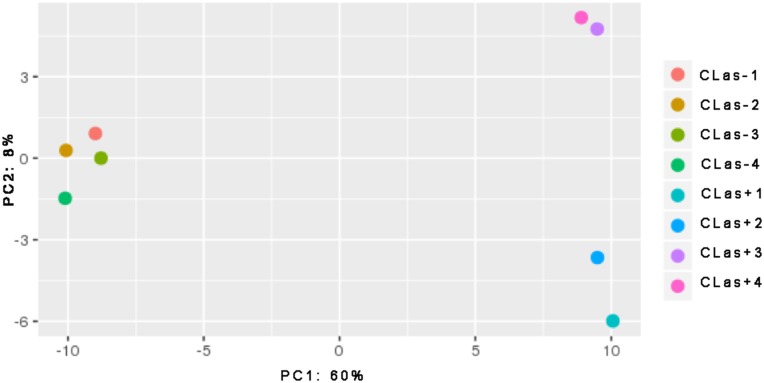
Principle components analysis (PCA) showing variations among replicates of RNA sequencing data. Four different replicates for each sample, either CLas exposed (CLas+) or non-exposed (CLas-, psyllids reared on healthy citrus plants) are plotted on the PCA plot. Approximately 60% of the variation is seen in principle component (PC) 1, which can be explained by differences in the CLas status of the samples (either + or -). Variation in PC2 is much less, 8% and can be ascribed to expression variation among CLas+ samples.

Illumina sequencing was performed on dissected guts from psyllids reared on either healthy (CLas-) or infected (CLas+) citrus plants. Out of 21,986 mRNA transcripts predicted in the ACP genome, 15,385 had read counts greater than one count per million in three or more replicates. These transcripts corresponded to 14,832 unique genes. Principal component analysis on the global dataset including all transcripts showed that 60% of the variation in the dataset could be ascribed to exposure to CLas (Fig 1). The second principal component had 8% variation in the replicates, which may be due to genetic differences in the ACP biological replicates or to technical variability (Fig 1). Variation in transcript expression was more pronounced in CLas+ samples, but this is not unexpected as not all insects from the CLas+ colonies test positive for CLas. The percentage of CLas+ ACP from these colonies was 82% with an average ct value of 31 for the infected insects, with ct values for individual insects ranging from 16 to 35 (S1 Fig). Control reactions containing no DNA have undetectable results using these qPCR conditions. A total of 965 transcripts were upregulated and 850 transcripts were downregulated at a 2-FC threshold (Table 1).

**Table 1:** Summary of transcriptome and proteome data and differential expression.

**Total transcripts**^1^	**Upregulated in CLas+**^3^		**Downregulated in CLas+**^3^	
14,832	965		850	
**Total Proteins**^2^	**ACP**^4^	**Microbiome**^5^	**ACP**^4^	**Microbiome**^5^
1,641	58	0	140	29

^1^ Number of transcripts identified by RNA sequencing with more than one count per million in three or more replicates

^2^ Number of proteins identified using proteomics with a minimum of two unique peptides

^3^ Number of transcripts or proteins with a p value of <0.05 after a Wald/exact test (transcripts) or Fisher’s exact test (proteins) and with a CLas+/CLas- Log_2_ fold change of ±0.5

^4^ Proteins matching to the *Diaphorina citri* proteome

^5^ Proteins matching to the proteome of *Candidatus* Carsonella rudii, *Candidatus* Profftella armatura, or *Wolbachia pipientus*

The proteome data included proteins from the insect as well gut-associated microbes. Out of a total 20,996 predicted genes, 1,641 proteins were unambiguously identified from 215,550 peptide spectra. The vast majority of proteins identified were psyllid, with a final count of 1521, and 120 proteins were of bacterial origin (Table 1). Of these proteins, 57 were upregulated due to exposure to CLas and all of these were ACP proteins (Tables 1, S1 Table). A total of 169 were downregulated, of which 29 were of bacterial origin, overwhelmingly from *Wolbachia* (Tables 1, 2 and S2 Table).

**Table 2:** *Wolbachia* proteins differentially expressed in CLas+ ACP guts.

Protein Annotation^1^	Protein GI^2^	Protein Accession^3^	Log_2_ Fold Change CLas+/-^4^	P Value^5^
iron deficiency-induced protein A	gi|516101257	WP_017531837.1	-3.170	0.033
Ankyrin	gi|516101663	WP_017532243.1	-3.044	0.047
membrane protein	gi|516101883	WP_017532463.1	-2.833	0.01
polynucleotide phosphorylase	gi|516101360	WP_017531940.1	-2.617	< 0.00010
50S ribosomal protein L1	gi|516101535	WP_017532115.1	-2.585	0.027
30S ribosomal protein S2	gi|648400952	WP_026092703.1	-2.077	0.0015
phosphoribosylamine—glycine ligase	gi|516101527	WP_017532107.1	-1.977	0.037
thioredoxin	gi|516101608	WP_017532188.1	-1.935	0.0015
membrane protein	gi|516101614	WP_017532194.1	-1.931	< 0.00010
30S ribosomal protein S6	gi|648400856	WP_026092607.1	-1.907	0.016
hypothetical protein, partial	gi|516101417	WP_017531997.1	-1.737	0.011
transcriptional activator protein	gi|516101552	WP_017532132.1	-1.585	0.02
elongation factor Tu	gi|516101903	WP_017532483.1	-1.570	0.00038
membrane protein	gi|516101809	WP_017532389.1	-1.439	0.024
hypothetical protein	gi|516101930	WP_017532510.1	-1.392	0.012
malate—CoA ligase subunit beta	gi|516101329	WP_017531909.1	-1.363	0.031
peptidoglycan-associated lipoprotein	gi|516101767	WP_017532347.1	-1.322	0.014
hypothetical protein	gi|516101366	WP_017531946.1	-1.170	0.039
hypothetical protein	gi|516101286	WP_017531866.1	-1.000	0.011
restriction endonuclease subunit S	gi|516101559	WP_017532139.1	-0.899	0.011
hypothetical protein	gi|516101179	WP_017531759.1	-0.802	0.035
molecular chaperone GroeL	gi|516101041	WP_017531621.1	-0.711	0.03
hypothetical protein	gi|516101398	WP_017531978.1	-0.636	0.049
succinyl-CoA synthetase subsunit alpha	gi|516101330	WP_017531910.1	Not found in CLas+	0.00014
membrane protein	gi|648400895	WP_026092646.1	Not found in CLas+	0.00078
elongation factor G	gi|648400891	WP_026092642.1	Not found in CLas+	0.0042

^1^ Annotation for corresponding protein/transcript from NCBI

^2^ Protein GI found in NCBI

^3^ Accession number for corresponding protein in NCBI

^4^ Log_2_ fold change generated by comparison of CLas+/CLas- spectral counts. A Log_2_ fold change cutoff of ±0.5 was applied for differential expression analysis

^5^ P-values were generated using a Fisher’s exact test and a p-value cutoff of <0.05 was applied

**Fig 2:**
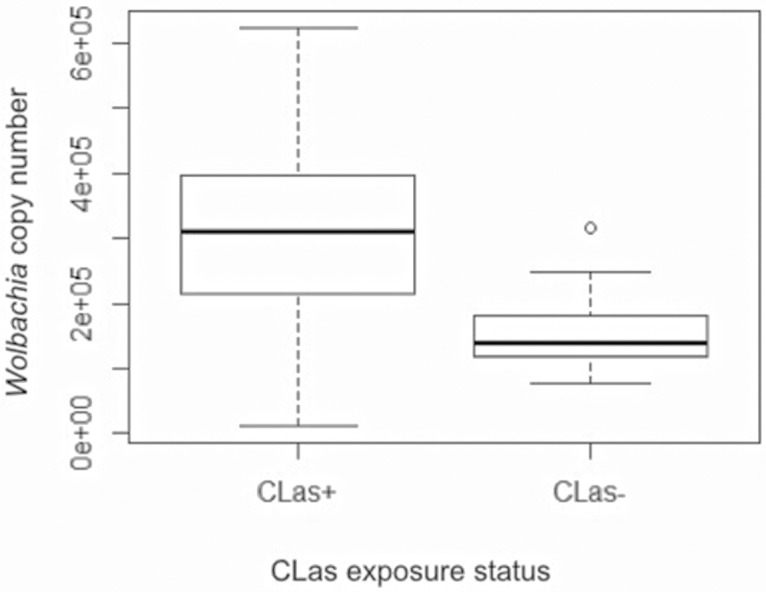
Single gut quantitative PCR showing *Wolbachia* copy number values in excised psyllid guts. Quantitative PCR was performed on DNA extracted from single, excised guts to determine if the titer of *Wolbachia* is altered in CLas-exposed (CLas+) as opposed to CLas-unexposed (CLas-) guts. The resulting copy numbers shown above are not statistically different using error bars reflecting the standard error of these calculated values. Black bars indicate the mean copy number, and the height of the boxes reflects the spread of copy number values.

An analysis of *Wolbachia* titer in the psyllid gut samples using qPCR showed that the levels of *Wolbachia* were not statistically different in CLas-exposed as compared to non-exposed insects (Fig 2). It is unlikely that the qPCR is detecting dead Wolbachia in this experiment as we can readily detect changes in Wolbachia titer when feeding ACP antibiotics through a liquid diet (Cilia, unpublished observations). These data show that exposure to CLas significantly alters the proteome of the psyllid gut and psyllid interactions with the gut microbiota without altering the titer of *Wolbachia*. Since CLas proteins were only present in CLas+ samples, they were not considered in the list of differentially expressed bacterial proteins.

From the 1521 psyllid proteins identified by mass spectrometry-based proteomics, 1428 were also identified by the RNAseq experiment. These 1,428 ACP proteins were identified on the basis of a minimum of two high scoring peptides. Only 44 ACP proteins had no corresponding transcripts in the transcriptome dataset; a number that likely resulted from the stringency we applied to the transcriptomic data read count. These include predominantly enzymes, including a glutathione transferase and a beta-lactamase, as well as core histone proteins such as histone H2A (S3 Table). The trend of up- and downregulation induced by CLas exposure was the same in the transcriptome and proteome for 101 genes. The transcriptomic and proteomic profiles showed antiparallel expression in nine genes; six proteins had corresponding transcripts detected as upregulated in the RNAseq data but downregulated in the proteome data (Table 3). Genes with mitochondrial function were well represented in this class (two of the six). Three proteins were downregulated in the RNAseq data but upregulated in the proteome data (Table 3).

**Table 3:** ACP proteins with antiparallel patterns of regulation between the transcriptome and proteome.

Protein accession^1^	Protein GI^2^	Annotation^3^	Log_2_Fold Change^4^ CLas+/CLas-	
			RNAseq	Proteomics
XP_008479572.1	662185929	collagen alpha-5(IV) chain	0.58	-2.87
XP_008467803.1	662190109	UDP-glucuronosyltransferase 2B7-like	0.60	-2.08
XP_008471666.1	662197199	putative tricarboxylate transport protein, mitochondrial	0.55	-2.11
XP_008477908.1	662208689	probable cyclin-dependent serine/threonine-protein kinase DDB_G0292550, partial	1.00	-2.46
XP_008478719.1	662210178	probable 3-hydroxyacyl-CoA dehydrogenase B0272.3	2.31	-1.17
XP_008479475.1	662211562	proline dehydrogenase 1, mitochondrial	0.60	-1.07
XP_008470971.1	662195929	vigilin-like	-0.52	0.86
XP_008471536.1	662196964	eukaryotic translation initiation factor 4B, partial	-0.78	0.65
XP_008485792.1	662223032	basic salivary proline-rich protein 2-like, partial	-0.62	1.31

^1^ Accession for corresponding protein in NCBI

^2^ Protein GI found in NCBI

^3^ Annotation for corresponding protein/transcript from NCBI

^4^ Number of transcripts or proteins with a p value of <0.05 after a Wald/exact test (transcripts) or Fisher’s exact test (proteins) and with a CLas+/CLas- Log_2_ fold change of ±0.5

### CLas exposure targets very specific aspects of ACP digestive biology

Trypsins, cathepsins, general proteases, and lipases were detected abundantly in the psyllid gut. Eight proteins annotated as cathepsin B and three proteins annotated as cathepsin L were detected in the proteome (Table 4). While most proteases were not differentially expressed upon exposure to CLas, one isoform of cathepsin B showed significant down-regulation (Table 4). Three additional cysteine proteases were upregulated in the transcriptome (Table 4). Ten other proteases and protease regulatory proteins were detected, as well as six lipases including one bile-salt activated lipase (Table 4). The transcriptome showed three lipases to be downregulated upon CLas exposure (Table 4). Mucins have also been implicated in normal digestion as well as defense against pathogens [41, 42]. Two mucins were detected in the proteome, and one (mucin 22) was upregulated in CLas-exposed guts (S1 Table). In the transcriptome, mucin 19 was found to be upregulated in CLas-exposed guts (S4 Table). A facilitated trehalose transporter, Tret1-like, which has the predicted function to facilitate the transport of the major sugar component of insect hemolymph, trehalose (α-d-glucopyranosyl-(1,1)-α-d-glucopyranoside) [43], was abundant and down-regulated in the transcriptome (log2FC = -0.64) and the proteome (log2FC = -3.39) in response to CLas (S2 and S4 Tables, respectively).

**Table 4:** Digestive proteins and transcripts detected in the ACP gut. Proteins annotated as proteases or lipases are included here from both the RNAseq and proteome dataset.

Protein Annotation^1^	Protein GI^2^	RNA/Protein Accession^3^	Log_2_Fold Change CLas+/-^4^
**RNAseq**			
putative cysteine proteinase CG12163 isoform X1	662190811	XM_008469966.1	3.053*
putative cysteine proteinase CG12163 isoform X2	662190813	XM_008469967.1	0.551*
putative cysteine proteinase CG12163 isoform X1	662190815	XM_008469968.1	0.331
cathepsin B-like cysteine proteinase 4 isoform X3	662196046	XM_008472814.1	0.692*
cathepsin B-like cysteine proteinase 4 isoform X3	662196048	XM_008472815.1	0.420
cathepsin B-like cysteine proteinase 4 isoform X3	662196052	XM_008472817.1	-1.141*
cathepsin B-like cysteine proteinase 4 isoform X3	662196054	XM_008472818.1	-0.761*
cathepsin B-like cysteine proteinase 4 isoform X3	662196056	XM_008472819.1	-0.385
cathepsin B-like cysteine proteinase 4	662203171	XM_008476687.1	0.357
serine protease 27-like, partial	662195706	XM_008472631.1	1.415*
venom protease	662198873	XM_008474353.1	1.158*
lon protease homolog, mitochondrial-like	662216156	XM_008483771.1	-0.829*
ATP-dependent zinc metalloprotease YME1 homolog, partial	662225174	XM_008488749.1	-0.612*
pancreatic triacylglycerol lipase-like	662197645	XM_008473689.1	1.017*
hepatic triacylglycerol lipase-like	662209497	XM_008480130.1	-1.607*
phospholipase A-2-activating protein-like	662211846	XM_008481403.1	-1.198*
**Proteome**			
lysosomal aspartic protease-like	662195753	XP_008470879.1	-0.518
26S protease regulatory subunit 6A-B	662187287	XP_008486389.1	-0.332
26S protease regulatory subunit 8	662196678	XP_008471380.1	-0.366
putative serine protease K12H4.7	662203334	XP_008475000.1	-0.807
26S protease regulatory subunit 10B	662206814	XP_008476881.1	-0.388
26S protease regulatory subunit 4-like	662207419	XP_008477211.1	-0.558
26S protease regulatory subunit 6B	662211003	XP_008479170.1	-0.133
protease Do [Wolbachia endosymbiont of Diaphorina citri]	516101422	WP_017532002.1	-1.044
ATP-dependent Clp protease proteolytic subunit, mitochondrial-like	662214431	XP_008481047.1	Not detected in CLas+
Cathepsin B-like cysteine proteinase 4 isoform X2	662196044	XP_008471035.1	-0.9*
cathepsin B-like	662203893	XP_008475303.1	-0.585
cathepsin L-like	662192665	XP_008469199.1	-0.366
cathepsin L1	662192931	XP_008469346.1	-0.755
cathepsin L1-like	662192635	XP_008469183.1	-0.364
cathepsin B-like	662196066	XP_008471047.1	-0.745
cathepsin B preproprotein-like protein, partial	110456454	ABG74712.1	-0.379
cathepsin B-like	662203391	XP_008475032.1	-0.585
cathepsin B-like cysteine proteinase 4	662203171	XP_008474909.1	-0.38
cathepsin B-like cysteine proteinase 4	662196064	XP_008471046.1	-0.763
cathepsin B-like cysteine proteinase 4	662189687	XP_008467581.1	-0.595
26S proteasome non-ATPase regulatory subunit 7	662189383	XP_008467415.1	0.889*
26S protease regulatory subunit 7	662198887	XP_008472582.1	0.222
26S proteasome non-ATPase regulatory subunit 12	662191282	XP_008468450.1	-3.229*
bile salt-activated lipase-like, partial	662212174	XP_008479807.1	-0.228
lysophospholipase-like protein 1	662199055	XP_008472675.1	-0.737
lysophospholipase-like protein 1, partial	662221428	XP_008484906.1	-0.807

^1^ Annotation for corresponding protein/transcript from NCBI

^2^ Protein GI found in NCBI

^3^ Accession number for corresponding protein in NCBI

^4^ Log_2_ fold change for transcript/protein comparing CLas+/CLas-

*Indicates a statistically significant difference between CLas+ and—at the protein or transcript level, with a P value <0.05 after a Wald/Exact test (transcripts) or Fisher’s exact test (proteins) and Log_2_ fold change cutoff of ±0.5.

Several proteins with predicted lysosomal functions were detected, but very few were differentially expressed. A GDP-mannose 4,6-dehydratase and a mannose-6-phosphate isomerase were detected in ACP guts (S3 Table). However, a gamma-glutamyl hydrolase A-like enzyme is predicted to be a lysosomal protease and was upregulated in CLas+ guts with a Log_2_ fold change of 1.0 (S1 Table). Gamma-glutamyl hydrolase B-like was upregulated in the transcriptome with a Log_2_ fold change of 1.18 (S4 Table). A lysosomal-trafficking regulator-like transcript was downregulated with a Log_2_ fold change of -0.53 (S4 Table).

In contrast to the lysosomal pathways, which appear to be buffered from the effects of CLas in the gut, transcripts and proteins associated with ubiquitin-mediated proteolysis were differentially expressed. Eight protein subunits of the 26S proteasome were identified, and two were downregulated (S2 and S3 Tables, respectively). Proteins involved in ubiquitin conjugation were also identified. The transcriptomic data detected five E3 ligases, of which three were upregulated in CLas+ guts and two were downregulated (S4 Table). One additional E3 ligase was identified in the proteome (S3 Table). There was a total of 16 proteins involved in ubiquitination differentially expressed in the transcriptome (S4 Table). While 15 ubiquitination proteins were identified in the proteome (S3 Table), only three were differentially expressed: small ubiquitin modifiers 1-like and 3-like, and ubiquitin carboxy-terminal hydrolase 5-like, which were all significantly upregulated (S1 Table). The comparison of transcriptomic and proteomic data implies that regulation of proteasomal subunits occurs on the post-transcriptional level, while regulation of proteins involved in the process of ubiquitination occurs at the transcriptional level.

### Insect immunity related genes are affected by CLas

Transcripts with well-characterized roles in host-pathogen interactions were differentially expressed in CLas-exposed ACP adult gut tissues. A transcript encoding a basement membrane-specific heparan sulfate proteoglycan [44] core protein showed three-fold downregulation in the transcriptome (S4 Table). A number of high affinity iron binding proteins including ferritin [45] were also detected in these data. A ferritin subunit was found to be upregulated in the proteome (S1 Table) and the transcriptome (S4 Table). Conversely, two transferrin-like transcripts were downregulated (Log_2_FC = -0.71, -073) (S4 Table). The proteins corresponding to these transferrin transcripts had even greater downregulation (Log_2_FC = -2.54, -1.17, respectively) (S2 Table). Two additional transferrin proteins were downregulated (Log_2_FC = -1.77, -1.15) (S2 Table). Synaptic vesicle membrane protein VAT-1, a transcript that is significantly upregulated in *Aedes aegypti* midguts exposed to Sindbis virus [46], was significantly upregulated in the ACP gut transcriptome (S4 Table). This transcript evidence is supported by proteomic evidence, which also showed upregulation of VAT-1, although it fell below our fold change cutoff for differential expression (S3 Table). A toll-like receptor 3 transcript was upregulated (S4 Table). We searched both datasets for proteins with leucine rich repeat (LRR) domains. Seven transcripts with leucine rich repeats were differentially expressed. Four were downregulated and three were upregulated (S4 Table). Five additional unique LRR-containing proteins were identified in the proteome (S3 Table). Of these, two were upregulated (S1 Table), two were not differentially expressed (S3 Table), and one was exclusively detected in CLas+ samples (S3 Table). ACP genes predicted to be involved in gene silencing annotated in [47] were considered, and from this list a regulator of nonsense transcripts homolog and a ubiquitin carboxy-terminal hydrolase were downregulated at the proteome level (Log2FC = -1.07, -1.39 respectively, S2 Table).

### Data show signatures of mitochondrial dysfunction and apoptosis in CLas exposed guts

**Fig 3:**
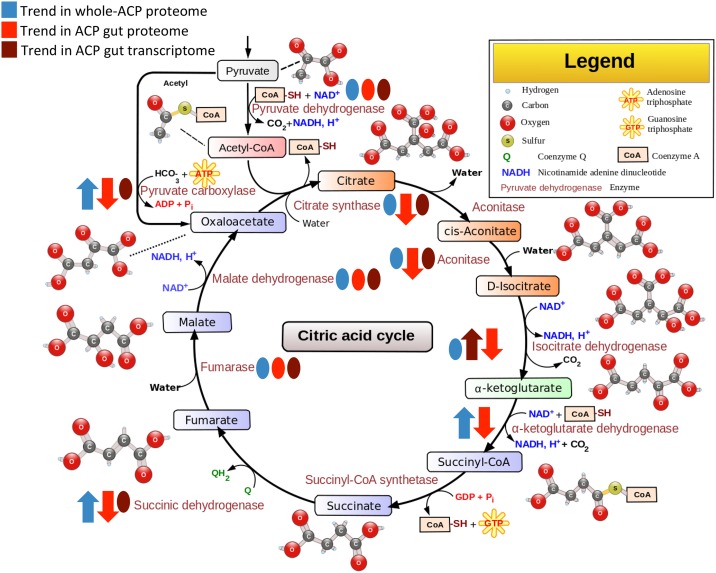
Schematic diagram of the citric acid cycle showing the trend of regulation in the proteome upon CLas exposure. This image was taken from https://en.wikipedia.org/wiki/File:Citric_acid_cycle_with_aconitate_2.svg and adapted to indicate up- and down-regulation shown by proteomic and transcriptomic data using the creative commons license http://creativecommons.org/licenses/by-sa/3.0/deed.en. Blue shapes reflect the whole body ACP proteome data from [14], red shapes reflect the ACP gut proteome, and maroon shapes reflect the gut transcriptome. Arrows indicate the trend of up- or down-regulation, and ovals indicate that these enzymes were detected but were not differentially expressed.

A total of 196 mitochondrial proteins were identified in the ACP gut proteome. Of these proteins, 26 were differentially expressed in CLas+ ACP (Table 5). Strikingly, 25 of these were downregulated upon exposure to CLas, showing a widespread depression of mitochondrial function. Several of these proteins were key enzymes in the citric acid (TCA) cycle (Fig 3). Pyruvate dehydrogenase, fumarase, and malate dehydrogenase were detected in both datasets, but not differentially expressed (S3 Table). Citrate synthase, aconitase, and pyruvate carboxylase were downregulated in the gut proteome (S2 Table) and detected in the transcriptome but not differentially expressed (data not shown). Antiparallel regulation occurred in some TCA enzymes. Two proteins annotated as isocitrate dehydrogenase were downregulated in the proteome (S2 Table), and one of them was upregulated in the transcriptome (S4 Table). Succinic dehydrogenase was also detected in the gut transcriptome, but not differentially expressed (Fig 3).

**Table 5:** Mitochondrial proteins differentially expressed in the ACP gut.

Protein Annotation^1^	Protein GI^2^	Protein Accession^3^	Log_2_FC CLas+/-^4^	P Value^5^
GTP:AMP phosphotransferase AK3, mitochondrial	gi|662212946	XP_008480235.1	-4.044	0.00081
NADH dehydrogenase [ubiquinone] iron-sulfur protein 3, mitochondrial-like, partial	gi|662225787	XP_008487314.1	-2.492	0.0027
putative tricarboxylate transport protein, mitochondrial	gi|662197199	XP_008471666.1	-2.109	0.021
isocitrate dehydrogenase [NADP], mitochondrial-like	gi|662186257	XP_008481256.1	-1.987	0.0055
electron transfer flavoprotein-ubiquinone oxidoreductase, mitochondrial-like	gi|662193641	XP_008469735.1	-1.977	0.037
heat shock 70 kDa protein F, mitochondrial-like isoform X1	gi|662219143	XP_008483637.1	-1.420	0.00028
very long-chain specific acyl-CoA dehydrogenase, mitochondrial	gi|662202858	XP_008474742.1	-1.354	< 0.00010
NADH dehydrogenase [ubiquinone] iron-sulfur protein 2, mitochondrial	gi|662187693	XP_008487487.1	-1.245	0.015
NADH dehydrogenase [ubiquinone] 1 alpha subcomplex subunit 10, mitochondrial	gi|662212934	XP_008480228.1	-1.242	0.0031
trifunctional enzyme subunit alpha, mitochondrial-like	gi|662196464	XP_008471263.1	-1.235	0.011
succinate dehydrogenase [ubiquinone] flavoprotein subunit, mitochondrial	gi|662188053	XP_008487682.1	-1.196	0.00018
probable hydroxyacid-oxoacid transhydrogenase, mitochondrial	gi|662185144	XP_008475568.1	-1.152	0.027
proline dehydrogenase 1, mitochondrial	gi|662211562	XP_008479475.1	-1.073	0.027
putative ATP synthase subunit f, mitochondrial	gi|662202808	XP_008474714.1	-1.073	0.019
isocitrate dehydrogenase [NADP], mitochondrial-like	gi|662186255	XP_008481246.1	-1.038	0.025
phosphate carrier protein, mitochondrial-like	gi|662191655	XP_008468651.1	-0.980	0.015
trifunctional enzyme subunit alpha, mitochondrial-like	gi|662196462	XP_008471262.1	-0.961	0.042
pyruvate carboxylase, mitochondrial-like	gi|662192943	XP_008469353.1	-0.931	0.0012
2-oxoglutarate dehydrogenase, mitochondrial	gi|662205659	XP_008476253.1	-0.911	0.024
4-aminobutyrate aminotransferase, mitochondrial, partial	gi|662191514	XP_008468575.1	-0.898	0.037
LOW QUALITY PROTEIN: glutamate dehydrogenase, mitochondrial-like	gi|662214999	XP_008481358.1	-0.892	0.01
probable aconitate hydratase, mitochondrial	gi|662199757	XP_008473057.1	-0.857	< 0.00010
3-ketoacyl-CoA thiolase, mitochondrial isoform X1 [Diaphorina citri]	gi|662207989	XP_008477522.1	-0.849	0.016
3-ketoacyl-CoA thiolase, mitochondrial isoform X2	gi|662207991	XP_008477523.1	-0.849	0.016
NADH-ubiquinone oxidoreductase 75 kDa subunit, mitochondrial, partial	gi|662194155	XP_008470014.1	-0.810	0.012
mitochondrial intermembrane space import and assembly protein 40-B-like	gi|662198120	XP_008472168.1	2.222	0.00021
ATP synthase subunit s, mitochondrial-like	gi|662200668	XP_008473556.1	**Not detected in CLas+**	0.00051
enoyl-CoA hydratase domain-containing protein 3, mitochondrial-like, partial	gi|662222663	XP_008485588.1	**Not detected in CLas-**	0.00057
LOW QUALITY PROTEIN: dynamin-like 120 kDa protein, mitochondrial, partial	gi|662191850	XP_008468756.1	**Not detected in CLas+**	0.0097

^1^ Annotation for corresponding protein/transcript from NCBI

^2^ Protein GI found in NCBI

^3^ Accession number for corresponding protein in NCBI

^4^ Log_2_ fold change generated by comparison of CLas+/CLas- spectral counts. A Log_2_ fold change cutoff of ±0.5 was applied for differential expression analysis

^5^ P-values were generated using a Fisher’s exact test and a p-value cutoff of <0.05 was applied

The only upregulated mitochondrial protein was related to inner membrane transport. This protein is annotated as a mitochondrial intermembrane space import and assembly protein 40-B-like (protein Log2FC = 2.222, Table 5, S1 Table). The transcriptome analysis enabled some additional insights into mitochondrial perturbations in the CLas exposed ACP gut tissues, namely that a mitochondrial cytochrome c oxidase subunit was significantly upregulated (Log2 FC = 2.89, S4 Table).

Beyond the observations implicating mitochondrial perturbation (Table 5, Fig 3) in CLas exposed ACP gut tissues and the cysteine proteases (Table 4), protein and transcript expression signatures of cell death were observed. A UV excision repair protein (Rad23 Homolog B-like) as well as a proteasomal factor (26S proteasome non-ATPase regulatory subunit 7) were upregulated in CLas+ samples (S1 Table). A total of 22 heat shock proteins were identified in the proteome, of which one came from *Wolbachia*, two from Profftella, and one from CLas. Four of these were downregulated, including one from Profftella. Nine heat shock transcripts were differentially expressed, of which four were upregulated and five were downregulated (S4 Table). Two unique thrombospondin family transcripts were found to be upregulated in CLas+ guts (S4 Table). Both were annotated as thrombospondin type-1 domain-containing protein 4-like. Thrombospondin is an extracellular glycoprotein receptor that catalyzes a cascade leading to apoptosis [48]. Two Annexin B12 transcripts were upregulated in the transcriptome (S4 Table). These observed changes may be related to trafficking perturbations and not apoptosis induction. Along that line of reasoning, the heavy and light chains of clathrin, and a phosphatidylinositol-binding clathrin assembly protein were detected (S3 Table). Calreticulin was slightly upregulated in the transcriptome, although it fell below the fold change cutoff applied, and co-filin/actin depolymerization homolog was downregulated in the proteome (S2 Table). No caspases were detected, although this could be a function of the incomplete annotation of the ACP genome or a low level of expression of these enzymes.

### Psyllids exposed to CLas show decreased expression of proteins associated with insecticide resistance in the gut tissue

Genes associated with insecticide resistance have previously been characterized in all the life stages of the ACP [49]. Psyllids harboring CLas have increased susceptibility to certain insecticides, including carbaryl, chlorpyriphos, fenpropathrin, imidacloprid, and spinetoram [50]. Consistent with this observation, we found that CLas-exposed guts show decreased expression of genes with potential roles in detoxification, including the cytochrome p450s and glutathione transferases (S2 and S4 Tables). These gene families have a diverse range of functions, but many members have a well-established role in insecticide resistance [51]. There was also a striking down-regulation of eight enzymes in the UDP-glucoronosyltransferase family (S2 Table), which have been associated with insecticide resistance and detoxification of compounds [52].

### Use of both transcriptomics and proteomics enabled complementary insights into the vector-pathogen relationship

The transcriptomic data revealed that two of the 30 most abundant transcripts in the ACP gut correspond to long non-coding RNAs (lncRNAs) found at LOC103514231 and LOC103513736. The function of these lncRNA molecules are not known, and their involvement in the vector-pathogen relationship would have gone unnoticed had we only conducted a proteomics analysis. BLAST analysis found no significant sequence homology to any annotated genes in NCBI. Furthermore, the lncRNA profile of the ACP gut appears to change significantly upon exposure to CLas. Differential expression analysis identified 83 lncRNAs with differential expression in CLas+ guts: 55 were upregulated in CLas+ guts and 28 were downregulated (S4 Table). Of these 83 sequences, only three had homologs in Genbank. The accession numbers for these transcripts were XR_541837.1, XR_541922.1, and XR_541972.1, with top BLAST hits from *D*. *citri* to Charged multivesicular body protein 2a-like (LOC103518178), transcript variant X2 misc_RNA, Uncharacterized LOC103519849 ncRNA, and ATP synthase lipid-binding protein, mitochondrial-like (LOC103521263) transcript variant X2 miscellaneous RNA, respectively.

In contrast, 120 proteins in the gut proteome were of microbial origin and their corresponding transcripts were not detected using the polyA enrichment strategy we used for transcriptomics analysis (Table 1). Very few microbial proteins were identified from Carsonella and Profftella, and even fewer were differentially detected. One Carsonella protein and five Profftella proteins were downregulated, and three Profftella proteins were detected in CLas- guts only (S2 and S3 Tables, respectively). Since these symbiotic bacteria normally reside in the bacteriocytes [7], it is possible that these proteins are the result of bacteriocyte contamination during gut sample preparation. However, the identification of *Wolbachia* proteins revealed a more complex interaction. A total of 85 *Wolbachia* proteins were identified in the psyllid gut (S3 Table). Of the 26 proteins differentially expressed, all were downregulated (Table 2). A qPCR analysis performed on single dissected guts with primers specific to *Wolbachia* showed that the mean *Wolbachia* copy numbers were not significantly different between CLas+ and CLas; however, *Wolbachia* copy numbers were statistically more variable in CLas+ guts (F test for equality of variances = 0.00127). These data show that there is proteome-level regulation of *Wolbachia* in the ACP gut tissue and that *Wolbachia* titer may change in response to CLas acquisition.

### Wolbachia and CLas reside in distinct subcellular compartments in the psyllid gut

**Fig 4:**
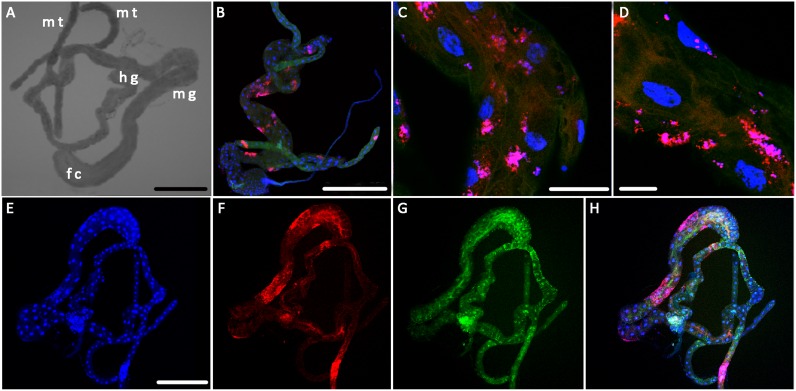
Fluorescent *in situ* hybridization to visualize microbial interactions in ACP guts. Dissected guts were reacted with probes specific to CLas (green) and *Wolbachia* (red) and imaged using confocal microscopy. Nuclei are stained using DAPI and visualized in blue. (A) Light imaging allowed for identification of regions of the gut, scale bar = 250μm, mt = Malpighian tubule, hg = hindgut, mg—midgut, fc = filter chamber. (B) Confocal micrographs of CLas- guts show no CLas signal and patchy distribution of *Wolbachia* (B, C, D), scale bars = 250μm, 50μm and 25μm, respectively. (E-H) Confocal microscopy of CLas+ guts shows a wider distribution of CLas in the gut and a patchy distribution of *Wolbachia* in the gut, with *Wolbachia* signal concentrated in the filter chamber, midgut, and Malpighian tubules, scale bar = 250μm.

**Fig 5:**
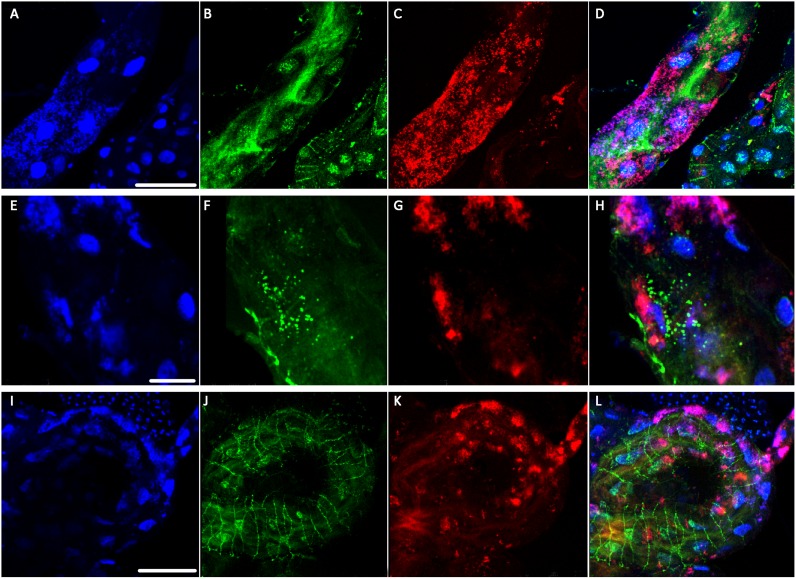
High magnification images of CLas and *Wolbachia* in CLas exposed guts visualized using fluorescence *in situ* hybridization (FISH) and confocal microscopy. CLas signal (Cy3) is in green, Wolbachia (Cy5) in red, and DAPI counterstaining of nuclei is in blue. (A-D) Optical cross-section of the gut visualizing CLas localization in along the brush border membrane of the gut lumen. Rarely, CLas can be seen co-localized with the DAPI signal, indicating nuclear association. *Wolbachia* signal does not overlap with CLas signal, see overlay (D), although the two bacteria are frequently observed within the same cell. (E-H) CLas can also be observed in puncta within cells and there is no overlap with Wolbachia in this distribution either. (I-L) At the basal surface of the gut, CLas is frequently observed along the actin cytoskeleton of the gut-associated muscles. *Wolbachia* signal is never detected in these filaments. Scale bars = A-D 25μm, E-H 25μm and I-L 75μm.

To confirm the proteome data indicating that *Wolbachia* is present in ACP gut tissue, we performed 16S sequencing from dissected ACP guts, and the PCR products aligned to *Wolbachia* sequences from other hemipteran insects (data not shown). To further characterize the relationship between *Wolbachia* and CLas in the psyllid gut, fluorescent *in situ* hybridization (FISH) was performed using a probe specific to *Wolbachia* and a probe specific to CLas (Figs 4 and 5). The ACP gut is divided into the foregut, midgut, and hindgut (Fig 4A). Guts that have not been exposed to CLas do not show a signal when the CLas probe is used (Fig 4B–4D). In unexposed guts from insects reared on healthy citrus plants, *Wolbachia* (red) appears to have a patchy localization in all regions of the gut, including the Malpighian tubules (Fig 4B–4D).

A total of 59 CLas exposed guts were examined using FISH. In CLas-exposed guts, CLas (green) was observed in all regions of the gut (Fig 4G). At the whole-gut level, *Wolbachia* had a widespread distribution throughout the psyllid gut tissue, including the midgut, filter chamber and Malphigian tubules, with the greatest signal in the midgut (Fig 4F). DAPI staining (blue) revealed that some gut cells displayed nuclear swelling and fragmentation of the heterochromatin (Fig 5A), as we have published previously [25]. At higher magnification, CLas was commonly observed along the brush border membranes of the gut epithelium facing the luminal surface as well as in the cytoplasm (Fig 5B). It was also frequently observed in distinct punctate loci within individual gut cells (Fig 5B and 5F). In the majority of CLas-exposed gut samples examined, CLas was localized along the gut-associated muscles (Fig 5J) to a structure we previously identified as the actin cytoskeleton using phalloidin [25]. *Wolbachia* assumed a distinct, patchy localization in the gut (Fig 5C, 5G and 5K) with diffuse localization within individual gut cells. Thus, CLas appears to change its association with subcellular structures depending on where in the gut it resides, whereas the localization of *Wolbachia* is restricted to the cytoplasm. The data show that *Wolbachia* and CLas are capable of residing in the same ACP gut cells, but do not have a high degree of co-localization within cells (Fig 5D, 5H and 5L).

### CLas is observed in ACP nuclei at a very low frequency

Very rarely, in 3/59 guts, CLas was observed associated with the nuclear membrane (Fig 5B and 5D) and nuclear association was not seen in every epithelial cell in gut samples where it was observed. A reconstruction of a 3-D confocal image series of the midgut shows that the CLas Cy-3 signal could be observed around the periphery of the nucleus, and possibly in the nucleus (S1 File). Wolbachia was not ever seen associated with the nucleus or along the actin skeleton of the gut-associated muscles (Fig 5C and 5G).

## Discussion

Transmission of plant and animal pathogens by insect vectors requires the pathogen to navigate a highly complex biological system [4]. The insect is a composite of diverse tissues, each with their own specific function. The gut faces some highly unique challenges such as CLas internalization into the gut epithelium and coping with membrane lipid damage occurring as a result reactive oxygen species produced by the CLas-infected tree [53–55]. Gut tissue-specific transcriptomics and proteomics provided us with a detailed look at what is happening at the insect gut interface at the proteome and transcriptome level. Since the biological replicates for the proteome and transcriptome experiments were collected over time, and extensive agreement in the signs of the differential expression was observed between the datasets, the two datasets served to validate one another. Our molecular-level understanding of psyllid-CLas interactions can only be as complete as the annotation of the genome. The genes in the transcriptome derived from the Diaci v1.1 genome using the NCBI Gnomon pipeline have been evaluated in Saha et al. (unpublished, preprint: http://biorxiv.org/content/early/2017/03/04/099168). An analysis using a set of 3550 Hemipteran clade-specific single copy and conserved genes found 74.7% to be complete and 25% missing (Saha et al., Supp Table 1). Therefore, there is a possibility that some of genes may be missing from this transcriptome and proteome analysis. This will be addressed in future work when an improved assembly and transcriptome are available.

One of the most striking observations of our study was impact of the CLas-infected tree on the TCA cycle in gut tissue. With the exception of sucinyl-coA synthetase, which was not detected in either this gut ‘omics analysis or a previous whole insect proteome analysis from our lab [14], enzymes regulating metabolic flux throughout the entire TCA cycle were downregulated in the gut. A comparison between the whole body [14] and gut-specific ACP proteomes (Fig 3) shows that the gut cells are experiencing a highly localized mitochondrial dysfunction which is not observed in other insect tissues. In fact, the quantitative whole body proteome analysis paints the opposite picture for enzymes carrying out the TCA cycle (Fig 3). In the whole body analysis, which includes proteomes isolated from the gut and all other psyllid tissues in a single homogenate, the trend was upregulation of pyruvate dehydronase, citrate synthase, aconitase, isocitrate dehydrogenase, fumerase, malate dehydronase [14]. However, we can now hypothesize that the changes were not statistically significant using the very stringent FC cutoff we selected in the whole body analysis [14], likely because the tissue-specific down-regulation of these enzymes in the gut (down) compressed the mean spectral counts in the whole body analysis. Upregulation of TCA cycle enzymes in other tissues may be a response to compensate for the localized mitochondrial dysfunction in the gut when insects are exposed to CLas-infected trees, and the gut may be buffering the rest of the ACP tissues from the destructive effects of CLas exposure or to toxic metabolites such as reactive oxygen species produced by the CLas infection in citrus host plants. This hypothesis is consistent with observations that CLas-exposed ACP do not have a fitness cost, and in fact are more fit and fecund [24, 26]. If CLas infection is damaging the gut as shown here and in a previous study from our lab [25], one question remains as to how infected insects cope with other pathogens. One possible explanation is that in the ACP’s highly-specialized phloem diet there is much less chance for sepsis because of a greatly reduced microbiome within the phloem. This is opposed to a chewing insect that assuredly consumes a much greater diversity of microorganisms from the leaf surfaces that could opportunistically infect damaged sites.

Other examples where tissue specific data provided unique insights can be seen in the analysis of the endosymbiont proteomes. At the whole insect level, changes in Profftella polyketide metabolism are observed [14] and almost no changes are observed in the *Wolbachia* proteome [14]. These data are in contrast to the downregulation of specific *Wolbachia* proteins observed in the psyllid gut. Our data show that *Wolbachia* is present in the ACP gut and undergoes proteome-level regulation in CLas+ guts. We also show that exposure to CLas causes an increased variability in *Wolbachia* copy number in individual gut samples. One hypothesis is that *Wolbachia* titer may be influenced by CLas titer in the gut. This hypothesis predicts that the variation in *Wolbachia* titer we observed in the CLas-exposed insects may be related to the high variability in CLas titer in individual ACP guts feeding on an infected plant (S1 Fig). *Wolbachia* is the most widespread insect symbiont, and the bacterium’s interaction with its host ranges from symbiotic to parasitic. The bacterium contributes to the amino acid synthesis in the insect host, and can influence sex determination in mosquito [56, 57]. *Wolbachia* is pertinent to the vector biology field, in particular as a mechanism to control mosquito transmission of animal viruses [58–60]. Proteomic and microscopic data presented here indicate a relationship between CLas and *Wolbachia* in which each bacterium occupies overlapping regions of the gut tissue, and regulation of *Wolbachia* occurs, in part, at the proteome level.

Both datasets revealed changes in ubiquitination ligases as well as proteases. While ubiquitination and proteolysis have not been well characterized in insect vector systems, proteins involved in ubiquitination have been identified as important factors in vector competency in *Aedes aegypti* and response to microbial attack in *Anopheles gambiae* [61, 62]. In *Sogatella furcifera*, two ubiquitin-related proteins (polyubiquitin and E3 ubiquitin ligase) were found to interact directing with a virus transmitted by the insect [63]. The nuanced regulation of proteasomal proteolysis in the ACP gut may be a pathway of interaction important to CLas transmission, particularly as it pertains to ACP interactions with *Wolbachia*. *Aedes albopictus* cell lines inoculated with *Wolbachia pipientis w*AlbB showed an increase in proteasome activity compared to un-inoculated cells [64]. The authors speculate that this increased proteolysis may increase the free amino acids available to *Wolbachia*. If such an interaction is also occurring between Wolbachia and the ACP, the release of free amino acids may allow CLas to use them preferentially, at the expense of *Wolbachia’s* nutrient availability. The assumption here is that CLas must have an alternative method of accumulating needed amino acids, perhaps a novel peptide uptake mechanism.

Insects lack the antigen-based immune pathways present in animals, and therefore must rely on innate immune responses such as cuticles, clotting, phagocytosis, encapsulation, and release of antimicrobial factors [65]. Insects such as *Drosophila* rely on four major immune pathways: IMD, JAK/STAT, Toll, and JNK. The genome of *Acyrthosiphon pisum* has recently been sequenced and shown to lack genes in the IMD pathway [66], and the IMD pathway genes are also notably absent from the transcriptome of the related, potato psyllid, *Bactericera cockerelli* [67]. No transcripts or proteins mapping to the IMD pathway were identified in our ACP dataset, although the ACP genome sequencing data are fragmented and the annotation of the genome is not complete [6, 32]. A previous transcriptomics analysis of the ACP also reported no transcripts belonging to the IMD pathway [47].

With no IMD pathway, hemipterans rely on alternative measures for immunity. CLas and the ACP have co-evolved to control CLas proliferation in the insect while not totally inhibiting CLas growth, but how? By acting on the expression of certain immune system pathways in the insect, selection has favored a low level of CLas proliferation in the insect vector at a level to ensure transmission without a significant fitness cost to the vector, another example supporting optimal virulence theory [68, 69] to explain the relationship between insect vectors and the pathogens they transmit. The differential regulation of heat shock proteins we observed in response to CLas may afford the ACP some flexibility to activate different components of its immune system, as heat shock proteins (HSPs) have been demonstrated to be an immune response to pathogens (including bacteria) and environmental stress in multiple insect systems [62, 70–73]. Exposure to CLas was found to significantly change the abundance of ferritin and transferrin, iron-binding proteins. Iron is an essential metal at the center of many host-pathogen interactions and selection has favored organisms to express highly complex mechanisms to sequester available iron. Ferritins have been implicated as antibacterial agents in other insect systems and are upregulated upon bacterial infection in mosquito, *Drosophila*, and silkworm [74]. They are thought to sequester iron from bacterial pathogens as a means of control. This hypothesis is consistent with the upregulation of ferritins observed in the ACP gut and provides a possible class of proteins with a role in the non-lethal limitation of CLas proliferation within the ACP. If limitation of iron is one way the ACP limits CLas replication, removing that limitation may result in an overproliferation of CLas and death of the vector. Intriguingly, *Wolbachia* may also be impacted by iron competition in the gut, as iron deficiency induced protein A showed the greatest downregulation in CLas-exposed guts.

Confocal imaging using FISH with CLas and *Wolbachia* specific probes enabled us to visualize where these microbes are located within the gut cells. CLas, but not *Wolbachia*, localizes to actin along the gut visceral muscle. In *Aedes albopictus*, the vector for Sindbis virus, Sindbis virus also localizes to the actin cytoskeleton of the visceral muscle tissues [75] Imaging also revealed a very low frequency of nuclear association of CLas; although other reports of FISH and immunolocalization of CLas within the psyllid guts, including from our own laboratory, have not observed this [25, 76]. In our laboratory, nuclear localization was not seen [25] until subsequent analysis of a larger number of sections and optimization of the analytical method. Due to the low frequency of this observation, it is possible that in other studies [25, 76, 77] nuclear localization or association was not noticed due to the smaller number of samples analyzed. Other explanations might include the use of different host plants to rear the ACP, differences in the labeling methods used, as well as different fixation methods used in these studies. A transient association with the nucleus would also make the observation more difficult. In any case, further analysis will need to be done to validate and further study this nuclear localization observation and to determine its role and importance in acquisition and transmission. Bacteria have been observed in the nuclei of the protozoan *Staurojoenina assimilis* within termite guts, and where they cause swelling of nuclei and eventual nuclear lysis and release of the bacteria [78]. Symptoms of nuclear swelling and lysis are observed in the CLas-exposed ACP gut (this work and [25]). However, presuming the low frequency at which we observe CLas associating with the nucleus using FISH is representative of its true distribution, the data do not explain the high frequency at which we observed nuclear swelling and fragmentation in gut epithelial cells [25].

Our FISH data showing nuclear fragmentation in CLas exposed guts are consistent with our previous report, and our OMIC’s data show increased expression of genes and proteins associated with apoptosis via the mitochondria, as well as stress. We observed increased expression of heat shock proteins as well as stress-associated detoxification enzymes. Our data expands upon previous work by presenting activation of mitochondrial cell death pathways as a possible mechanism for the induction of apoptosis in the gut. The major research question that follows is whether apoptosis and mitochondrial impairment in the ACP gut is a response of the insect to a) ROS and other phytotoxins produced by the CLas infected host plant and hence independent of CLas invasion into the gut tissue, b) a bacterial invader perceived as a pathogen, or c) a mechanism for the bacteria to increase its dispersal from the gut into the ACP hemolymph. If this is indeed an immune response, the psyllid’s response may explain why adult insects cannot efficiently acquire CLas [79]. The immune response hypothesis is also in line with studies that show the relationship may be mutually beneficial for CLas and the ACP: CLas enhances ACP reproduction [26], influences dispersal behavior, flight capacity and sexual attraction to mates [80]. Individual ACP find CLas infected trees more attractive initially, but once infected with CLas, insects find healthy trees more favorable [81].

Our data provide insights into the biological question of the propagative nature of CLas in the ACP. Recent reports indicate that CLas titer can increase in the psyllid, independent of a host plant [22]. Other vector-pathogen pairs provide context for the effects of a propagative pathogen on its insect vector. In the case of *Tomato spotted wilt virus*, the virus induces an immune response in its insect vector, the western flower thrip [82]. Furthermore, propagative viruses induce apoptosis in their insect vectors. *Rice ragged stunt virus* causes apoptosis in the salivary gland of its insect vector, the brown planthopper, and apoptosis increases viral transmission [83]. West Nile virus, which replicates in its mosquito vector, causes apoptosis in the midgut cells [84]. Our observation of immune system activation, mitochondrial dysfunction, and hallmarks of apoptosis at the transcript and protein level are consistent with impacts on insect vectors by propagative pathogens. However, it is pertinent to note that CLas is unculturable, and a test of the hypothesis that CLas directly induces apoptosis in the psyllid gut would be difficult. An alternative explanation is that the gut is damaged by phytotoxins produced in the phloem sap of CLas-infected trees.

The data reveal important information regarding the future development of new HLB management tools. As has been previously suggested [85], ACP proteins involved in digestion are of particular interest. Both datasets identified abundant enzymes associated with normal digestion in the ACP gut, including proteases, cathepsins, and lipases. The most highly expressed proteins in the gut are digestive proteases where the function is inferred based on high level of expression. Other than a report on cytochrome P450s, glutathione transferases and recombinant ACP cathepsin B [85], little to no enzymology or cell biology has been performed to determine localization of activity of ACP digestive enzymes. Given that insects have such diverse physiologies, experiments should be performed to determine whether gut enzymes are digestive and/or lysosomal in the ACP. A facilitated trehalose transporter was also potently downregulated in both datasets. Trehalose is the most abundant sugar in insect hemolymph, and is the main source of energy and carbon for these organisms. It is a disaccharide formed by α (1, 1) glucoside linkages. It plays an important role in anhydrobiosis as well as other environmental stress responses in insects and other arthropods [43, 86]. In each ACP gut dataset, the trehalose transporter Tret1 was among the most differentially expressed. This observation indicates a fundamental change in gut trehalose metabolism upon exposure to CLas. Furthermore, it has been observed that changes in the membrane environment of cells alter the activity of trehalose [87]. Trehalose is known to affect the pH of insect hemolymph and therefore changes in hemolymph pH as a function of trehalose concentration would be expected to have an impact on the structure and function of enzymes. Trehalose transport has been shown to increase at low pH [88]. The increased transport evidenced by the differential expression of the tret1 transcript and protein indicates that ACP hemolymph and/or gut pH may be affected by exposure to CLas.

Two novel long lncRNAs were identified by the transcriptome and were highly differentially expressed upon CLas exposure. LncRNAs are transcribed by RNA pol II and are often spliced, capped, and polyadenylated much like mRNAs, which allowed them to be identified by the enrichment for polyadenylated RNAs. Their expression is induced by numerous environmental and developmental stimuli, including heat shock and embryogenesis. In other systems, lncRNAs function as regulators of apoptosis and inflammation, as well as in developmental regulation [89, 90]. Accumulation of lncRNAs has been associated with intracellular bacterial infection in *Salmo salar* [91]. Some lncRNAs have even been associated with insecticide resistance [92] and the proteome data illuminated that proteins regulating insecticide detoxification were downregulated in the gut tissues of CLas-exposed insects. Much work is being done to characterize lncRNAs, but little is known about their function to date. The minimal information available indicates their essential role in responses to stress as well as normal lifecycle in insects, so this class of molecule may prove to be a novel target for vector control in the HLB pathosystem, for example by delivering antisense molecules to insects, in planta, targeting these sequences using the citrus-infecting *Citrus tristeza virus* [93].

## Supporting information

S1 Fig Frequency histogram representing the number of individual psyllids at each ct value measured from a CLas positive colony. Ct values were obtained using quantitative PCR (qPCR) to assess CLas titer performed on individual adult psyllids feeding on a CLas-infected plant. Ct values show variation from 16 to 37. Ct values above 35 are considered to be CLas-negative.
(JPG)

S1 File A reconstruction of a 3-D confocal image showing CLas association with psyllid gut epithelial cell nuclei. A representative image is shown. CLas is imaged in green (Cy3), *Wolbachia* in red (Cy5) and DAPI staining is shown in blue.
(AVI)

S1 Table A list of all proteins upregulated in the ACP gut in response to CLas. A Fisher’s exact test was used to compare nLC-MS/MS spectral counts between treatment groups, and a P-value cutoff of 0.05 was employed to determine significance. A log_2_ fold change cut off of 0.5 was applied for differential expression.
(XLSX)

S2 Table A list of all proteins downregulated in the ACP gut in response to CLas. A Fisher’s exact test was used to compare nLC-MS/MS spectral counts between treatment groups, and a P-value cutoff of 0.05 was employed to determine significance. A log_2_ fold change cut off of -0.5 was applied for differential expression.
(XLSX)

S3 Table A list of all proteins detected in the ACP gut using nLC-MS/MS. Tandem mass spectra were matched to peptide sequences in a custom database containing endosymbiont, CLas and psyllid proteins at a 95% threshold with a 0.03% decoy false discovery rate (FDR). Proteins were identified with a 99% threshold and 0.6% decoy FDR with a minimum of two matching peptide sequences.
(XLSX)

S4 Table A list of all transcripts differentially expressed in the ACP gut in response to CLas exposure. Transcripts were considered differentially expressed using a log_2_ fold change cut-off of 0.5 and a false discovery rate (FDR) of < 0.01. A negative binomial Wald test was used with the FDR threshold of 0.01 and a Benjamini-Hochberg adjusted p-value threshold of 0.05. An exact test for differential expression for negative binomially distributed counts was implemented in with the same FDR threshold and fold change cut-off. Details on the software tools for the analysis are described and referenced in the Methods section.
(XLSX)
