# Supplementary figures and images for: Combining 'omics and microscopy to visualize interactions between the Asian citrus psyllid vector and the Huanglongbing pathogen Candidatus Liberibacter asiaticus in the insect gut
Source: PLoS One. 2017 Jun 20;12(6):e0179531. doi: 10.1371/journal.pone.0179531 (PMC5478155; doi:10.1371/journal.pone.0179531)

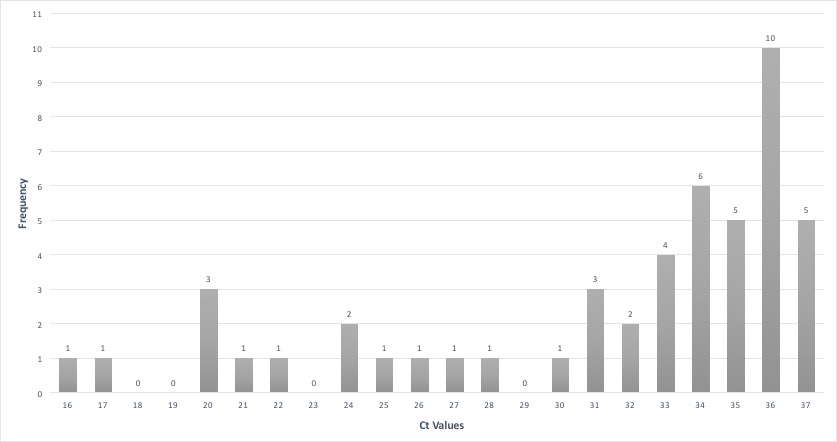

Supplement: S1 Fig — Ct values were obtained using quantitative PCR (qPCR) to assess CLas titer performed on individual adult psyllids feeding on a CLas-infected plant. Ct values show variation from 16 to 37. Ct values above 35 are considered to be CLas-negative. (JPG) [file pone.0179531.s001.jpg]
